# Radio Astronomical Antennas in the Central African Region to Improve the Sampling Function of the VLBI Network in the SKA Era?

**DOI:** 10.3390/s22218466

**Published:** 2022-11-03

**Authors:** Marcellin Atemkeng, Patrice Okouma, Eric Maina, Roger Ianjamasimanana, Serges Zambou

**Affiliations:** 1 Department of Mathematics, Rhodes University, Artillery Rd, Grahamstown 6139, South Africa; 2 Gabon Space Agency, Libreville P.O. Box 3850, Gabon; 3 Department of Physics and Electronics, Rhodes University, Grahamstown 6139, South Africa; 4 Instituto de Astrofísica de Andalucía (CSIC), Glorieta de la Astronomía, E-18008 Granada, Spain; 5 Semiconductor Development Division, Beneq Oy, Olarinluoma 9, 02200 Espoo, Finland

**Keywords:** radio astronomy, square kilometre array, very long baseline interferometry, radio interferometer

## Abstract

On the African continent, South Africa has world-class astronomical facilities for advanced radio astronomy research. With the advent of the Square Kilometre Array project in South Africa (SA SKA), six countries in Africa (SA SKA partner countries) have joined South Africa to contribute towards the African Very Long Baseline Interferometry (VLBI) Network (AVN). Each of the AVN countries aims to construct a single-dish radio telescope that will be part of the AVN, the European VLBI Network, and the global VLBI network. The SKA and the AVN will enable very high sensitivity VLBI in the southern hemisphere. In the current AVN, there is a gap in the coverage in the central African region. This work analyses the increased scientific impact of having additional antennas in each of the six countries in central Africa, i.e., Cameroon, Gabon, Congo, Equatorial Guinea, Chad, and the Central African Republic. A number of economic human capital impacts of having a radio interferometer in central Africa are also discussed. This work also discusses the recent progress on the AVN project and shares a few lessons from some past successes in ground stations retrofitting.

## 1. Introduction

The spatial resolution of a particular telescope determines how well one can see the details of cosmic objects. This resolution depends on the size of the telescope and the wavelength of the astronomical sources. To have a better resolution, one solution is to build a telescope with a large diameter. However, there is a practical limitation on the size of a telescope, and this led to the development of interferometry. That is, instead of having one large telescope, one can cross-correlate signals from individual antennas, and the resolution of the combined array of the antennas (the so-called interferometer) is determined by the largest separation (baseline) between the individual antennas. The combined array is therefore equivalent to a large single-dish telescope with a diameter equal to the longest baseline. Many radio telescopes were built after the development of the interferometry techniques. However, by the mid-1960s [1], it was realised that some radio sources could not be resolved even with radio telescopes of a few hundred km baselines. The quest for higher resolution led to the development of the Very Long Baseline Interferometry (VLBI) [2]. The VLBI is a technique of cross-correlating signals recorded by different antennas (and/or an array of antennas) separated by a large distance of up to the diameter of the earth. With this technique, detailed images of astronomical objects at milliarcsecond resolution have been obtained. In addition, high-precision astrometry has also been achieved.

South Africa and Australia are currently leading one of the most powerful and modern radio telescopes, the Square Kilometre Array (SKA) [3]. The SKA will be split in a mid-frequency (350 MHz–14 GHz) part build in South Africa, which will incorporate the MeerKAT telescope [4], and a low-frequency (50–350 MHz) part in Australia. To enable high-resolution interferometry through the VLBI, the SKA South Africa currently leads an effort to convert existing unused telecommunication dishes in partner countries (Botswana, Ghana, Kenya, Madagascar, Mauritius, Mozambique, Namibia, and Zambia) to radio telescopes. The converted antennas will then become part of a network of antennas distributed throughout Africa to form the African VLBI Network (AVN) [5]. Ghana has already successfully converted its old telecommunication dish to a working radio telescope [6]. Efforts to do the same in other African partner countries are underway. The AVN will significantly improve the science capabilities of the global VLBI community [7]. The AVN combined with the existing international VLBI facilities will produce huge quantities of data, presenting new challenges in data processing and storage. New techniques to manage the data must be developed, including: storage systems and data compression techniques; machine learning methods; software design techniques, control, and monitoring systems that parallel the internet of things; data flow architecture; and systems dealing with massive-scale computing. All of these challenges will strengthen the scientific collaborations between South Africa and its partner countries. In addition, Africa will become an international science and technology focus.

The central African states are currently not part of the AVN. This paper investigates the technical impact of this in terms of the AVN image quality and science capabilities. We will demonstrate by means of simulations how the AVN image signal-to-noise ratio (S/N) improves if antennas were added in these countries. This paper also highlights the economical and technological benefits for these countries should they join the AVN project. The main objective of this work is to stimulate a discussion within the global VLBI community on the need for additional antennas in the central African region.

## 2. Motivations

The Central African States (ECCAS—Economic and Community of Central African States) is a group of countries located in the centre of the African continent. These countries are located along the equator, have an ideal position, with a tropical climate, constant duration between days and nights, and no harsh climate, hence being ideal for observation. The ECCAS countries are mostly French speaking, with little use of English, and their integration to world scientific projects has been significantly slower compared to that of other African countries. Except for Cameroon, the countries in that space rank at the bottom of all continental and worldwide scientific metrics. In Africa, most scientific projects are split/shared between the North and the South. Northern African states are very close to Europe, hence they enjoy the benefit of that proximity in terms of scientific collaboration and industrial outsourcing. Southern African states are mostly English speaking and slightly wealthier than other parts of the continent, hence they have been very successful in attracting world-class scientific projects to their area. A similar trend is observed in the East and West African countries which have a large number of English-speaking countries. The ECCAS states are lagging, and the low scientific output, in terms of projects and journal publications in the region, correlates these facts [8]. To date, the ECCAS have not joined the AVN, even though significant advantage could open up for the region by becoming part of the AVN. Moreover, due to their geographical locations, the existing AVN community will significantly benefit from the participation of that region. Below are some non-exhaustive benefits of joining the AVN.

### 2.1. Education and Research Impacts

Joining the AVN will boost international cooperation in the field of astronomy and engineering and enable participation in international scientific research. Running a radio telescope requires skilled engineers, scientists, and technicians who will manage and run the facility. These personnel need to be trained in various disciplines, from radio astronomy and astrophysics to computer science and engineering. The decision to join the AVN network will trigger the development of critical skills and the institutional capacity necessary to optimise the ECCAS participation in the SKA. The high-end technologies and high-performance computing facilities needed to operate and maintain the telescopes are being developed in South Africa. The Centre for High-Performance Computing (CHPC) is already in place and running.

Ghana has already successfully converted its old telecommunication dish into a functioning radio telescope [6]. This is a demonstration that Africa can participate in high-level scientific research. The skills and experience from the development of these facilities will be transferred to the ECCAS if they join the AVN project. In addition, the project will bring new scientific opportunities to the ECCAS countries in a relatively short timescale. Currently, students in the AVN partner countries are benefiting from a number of scholarships to pursue further studies, train, and acquire more skills. Some of those scholarships include but are not limited to: the South African Radio Astronomy observatory (SARAO) scholarship; the Development for Africa with Radio Astronomy (DARA) project in the United Kingdom; and a number of South African Research Chairs Initiative (SARChI) of the Department of Science and Technology and the National Research Foundation. Another benefit in joining the project includes the training of African scientists, engineers, and technicians, with a view to ensuring that partner countries benefit from the second phase of the SKA. Along this issue of human capacity consolidation, a number of surveys have already highlighted the relatively weak hands-on exposure to core engineering and artificial intelligence skills among graduates trained in mathematical sciences in Africa. Towards possibly mitigating this diffuse pathology in the African Higher Education ecosystem, it is worth noting that a growing number of open-source hardware (and software) platforms offer, today, compelling tools for early exposure to what we could naively term the applied component of mathematical sciences. Due to its cross-discipline nature, radio interferometry has long been recognised as a suitable operational framework for impacting both conceptual and practical aptitudes to graduates. This can be illustrated by a pathfinding teaching and learning approach jointly adopted by Otago University (New Zealand), Rhodes University, and Stellenbosch University (South Africa) in deploying three mature versions of the Transient Array Radio Telescope (TART). TART (https://en.wikipedia.org/wiki/Transient_Array_Radio_Telescope accessed on 15 June 2022, https://www.womeninscience.africa/newly-launched-radio-telescope-opens-up-stem-learning-opportunities/ accessed on 15 June 2022) is an inexpensive open-source 24-elements radio telescope specifically designed to be a platform for experimental imaging algorithms for undergraduate and graduate students as well as researchers. It has the unique feature to allow the filling of multi-scale gaps in a value chain ranging from a basic knowledge of a diode to the most advanced mathematics embedded in artificial intelligence and related fields [9]. TART holds the range of versatility and affordability suitable for various intensities and targets for training African graduates in aperture synthesis as a way to better integrate a number of them into the AVN and SKA era.

### 2.2. Economic Impacts

The AVN will trigger foreign investment and expenditure (including local contracts and suppliers). The skills development that will be promoted by the project should enhance ECCAS’ engineering and scientific capabilities, promoting science and engineering breakthroughs for other sectors, such as medicine, remote sensing, and telecommunication, thereby enhancing innovation and competitiveness among industries. The construction or conversion of the telescopes will pave the way for a major boost to the local businesses, e.g., tourism industries, construction industries, and hospitality industries, thus creating new job opportunities and enhancing local revenue.

### 2.3. Scientific Impacts and Capabilities

Each AVN antenna will be designed to work as a single-dish radio telescope and as part of a larger VLBI network. The Ghana Radio Astronomy Observatory (GRAO) can serve as a reference case of what would be the science capabilities of the AVN antenna. The GRAO currently operates at 6.7 and 5 GHz frequencies. Future bands potentially include the L-band and the K-band receivers. Thus, as a single dish, it can be used to detect methanol masers, water masers, and pulsars; perform spectral line imaging; and observe continuum sources from AGNs and star-forming regions. The first detection of the methanol maser, G9.621+0.196E, was successfully performed by the GRAO on 21 November 2016. In addition, the GRAO detected the Vela pulsar (PSR J0835-451) at 5 GHz on 30 April 2017 [10]. In addition, the GRAO is currently being used to promote astronomy outreach [11] and was also used to detect the planet Mercury as it transited in November 2019 [12]. As part of a VLBI network, the AVN has a range of applications. This includes astrometry, geodesy, spacecraft tracking, and high-resolution imaging of distant radio sources. When combined with the global VLBI network worldwide, the AVN will significantly improve the imaging capability of the current VLBI network by providing more arrays and longer baselines.

### 2.4. Recent Progress on the AVN Project

The latest developments in the AVN project in each of the partner countries were reported during the 6th SKA Africa Partner Countries Meeting from 14 to 17 October 2019 in Pretoria, South Africa. As reported by South Africa’s Department of Science and Technology (DST), capacity building was one of the top priorities for each of the AVN partner countries. For example, through the two-dish interferometer concepts, universities in partner countries will be equipped with small radio telescopes aimed at radio astronomy training and astronomy outreach activities. The Botswana International University for Science and Technology already secured its two-aerial dish interferometer, UK Newton Funding, and a 10-PC laboratory to train students in radio astronomy. In this regard, Kenya also reported during the meeting that it plans to set up its two-dish interferometer in the near future. Training in the field of high-performance computing and big data is also underway, as presented in Section 2.5.

### 2.5. Scientific and Technical Training

The DARA project is intended to develop technical and scientific skills in a number of African countries, targeted to form the AVN. This program consisted of four training modules that were provided to 10 students from three countries, namely Zambia, Kenya, and Namibia, and four more AVN partners, which are Botswana, Ghana, Madagascar, and Mozambique. The JUMPING JIVE WP9 collaborated with the DARA project in a number of ways. From its pool of scientific and technical expertise, the JUMPING JIVE WP9 incorporated necessary skills into the already existing DARA network. More specifically, the JUMPING JIVE WP9 mobilised and supported European Union experts and scientists to tutor, advertise, and develop an interest in astronomy within the AVN partners. The JUMPING JIVE WP9 also allowed for African personnel to visit or have placements at European institutes as well as setting up an AVN technical support forum. Some of the organisations involved in providing personnel from the UK are the universities of Leeds, Oxford, Manchester, Central Lancashire, and Bristol. South African institutes and universities, including the SARAO, also contribute to the DARA project. In order to develop the technical and scientific skills necessary for the ECCAS to join the AVN network, it would be necessary for the DARA to extend its network into the ECCAS and initiate training programs in these countries.

## 3. Background and Past Successes on Ground Stations Retrofitting

In this section, we discuss some currently abandoned telecommunication antennas in the ECCAS region and present the requirements for a station to join the AVN, then discuss lessons learned from past successes on ground stations retrofitting.

### 3.1. Existing Abandoned Telecommunication Facilities

**Figure 1:**
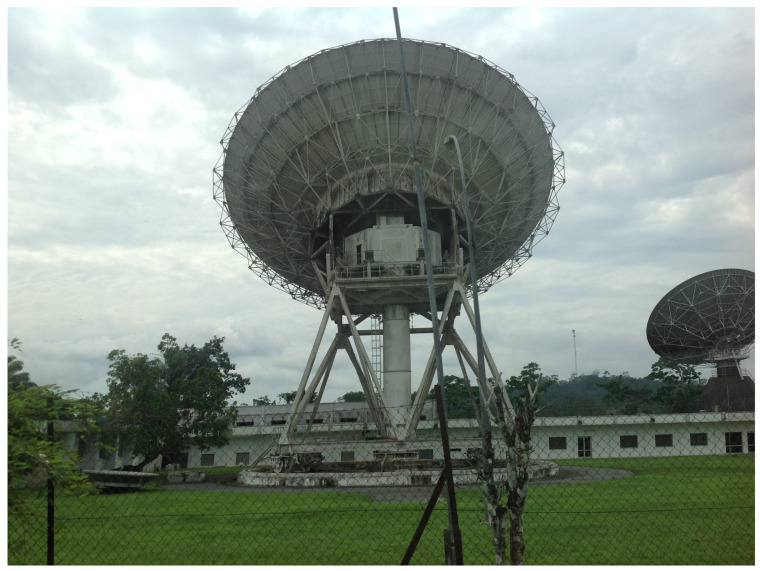
Decommissioned 32-m large satellite earth station antennas in Gabon.

The potential benefit of converting ground stations for radio astronomy has been recognised worldwide. The SKA Africa partner countries that currently host such redundant large antennas are South Africa (3 antennas), Ghana, Kenya, Madagascar, and Zambia. Similar facilities have been located in Algeria (2), Benin, Cameroon (2), the Congo Peoples Republic, Egypt (2), Ethiopia, Malawi, Morocco, Niger, Nigeria (3), Senegal, Tunisia, Uganda, Gabon (2), and Zimbabwe. As illustrated in Figure 1, Gabon is a country in the ECCAS community currently hosting such an idling ground station. This station is typical of others in the ECCAS region. It was of the type commissioned between 1970 and 1985, as a node of access into the global network of the Intelsat Satellite Earth Stations. The radio bands allocated for these satellite communications were typically 5.925–6.425 GHz for the up-link and 3.700–4.200 GHz for the down-link. For optimal operations, these antennas had to be at least 30 m in diameter [5]. Today, while remaining the property of the state, the idling dish in Figure 1 is part of the infrastructure currently leased to a private operator in the mobile telephony sector. It is located in an area known as Nkoltang, in the Northern part of Libreville. The dish is adjacent to the telemetry ground station used by the French CNES to perform follow-up of the ARIANE launch from French Guyana. If refurbished, idling satellite earth station antennas such as the one in Gabon will operate from existing facilities relatively close to cities in a relatively populated neighbourhood. The issue of a relatively high level of pre-existing Radio Frequency Interference (RFI) will therefore need to be dealt with appropriately. Standard procedures currently in place in Kuntunse easily serve as a template. Kuntunse, in Ghana, is currently the site hosting the first ever idling ground station in Africa that has been successfully refurbished to become an operational radio telescope. In the absence of dedicated protocols, the dominant approach there consists of performing standard flagging during the data analysis with standard packages. Such an approach has the advantage of providing further opportunities for building capacity in standard procedures, using standard resources often very well supported by the community. Coupled with the advent of the MOOC (Massive open online course), the initial demand on expert human capital as trainers is therefore minimised.

Given their relative availability on the African continent, the case for ground stations retrofitting raises two points of interest that we explore in the next sections. Namely, (1) what is required for a typical station to join the AVN and (2) what are some of the lessons learned from previous success stories of ground stations retrofitting.

### 3.2. Minimal Requirements for a Typical AVN Station

We use the GRAO as a benchmark for a typical AVN station. The GRAO is a former telecommunications satellite earth station whose initial conversion phase was concluded in August 2017. It was in part funded by South Africa and the African SKA partnership agreement. The GRAO is the first station of the intended network of radio telescopes, on the African continent, to support the existing VLBI. In the African context (excluding South Africa), and given the effort already spent on it, we conservatively base indications for minimal specifications for joining the AVN on the GRAO ones listed below. We base most of this section on [6,10,13].

With a primary 32 m diameter for its reflector and an estimated aperture efficiency of 0.5, the GRAO can achieve a System Equivalent Flux Density (SEFD) of 860 Jy using an ambient (uncooled) receiver at a temperature of 125 K. The switch to a cryogenically cooled receiver was assessed to be able to provide a decrease by a factor of 5 in the GRAO’s SEFD [13]. The GRAO has a fully steerable, Cassegrain beam-waveguide design dish. It is connected to a room temperature receiver and is currently capable of observing at 5 and 6.7 GHz (C band) with a bandwidth of up to 400 MHz. Although still short of funding, the current intentions are for the future acquisition of a 1.4–1.7 GHz (L-band) receiver. With further funding, subsequent plans are to replace the original C-band feed horn with a wider band design covering more VLBI bands and introducing cryogenic receivers for improved sensitivity [6].

As the VLBI observations require coherent per-baseline sampling while on target, the timing and frequency reference considerations suggested the choice of a hydrogen maser frequency reference, on-site, for the GRAO. Although the choice of such a frequency reference comes with issues of sensitivity to vibrations, thermal and magnetic field changes, this provides a benchmark that could be systematised for other facilities of a similar nature on the continent. At the GRAO, the VLBI backend is supported by the standard EVN hardware comprised of a Digital-Baseband Convertor (DBBC) and Mark-5b data recorder. A data archiving system using an HDF5 container and data-visualisation software was adopted as a standard for the GRAO [13,14].

The e-VLBI mode of operation consists of transferring data over a suitable link directly from the station to the correlator. It is a modern departure from the standard VLBI data transfer via physical disks shipping. In the e-VLBI mode, the required data rate (in Megabits per second) is given by $\Delta \nu \times n_{\mathrm{pol}}\times n_{\mathrm{bit}}\times 2$, where Δν is the bandwidth in Megahertz, $n_{\mathrm{pol}}$ is the number of observed polarisation, and $n_{\mathrm{bit}}$ is the number of bits per sample. A 128 MHz observing frequency would require 1024 Mbps connectivity to support a dual-polarisation e-VLBI mode observation using 2-bit sampling [13]. Presently, at the GRAO, internet connectivity to support the e-VLBI mode is not available yet.

### 3.3. A Few Lessons from Some Past Successes in Ground Stations Retrofitting

Ghana is uniquely located at 5 deg north of the equator. This allows viewing the entire plane of the Milky Way galaxy and nearly the whole sky, thus suggesting potentially compelling science cases. Already in 2017, the very first VLBI fringe tests were performed. These tests were satisfactorily conducted with the C-band (3.8–6.4 GHz) communication receiver. The outputs were clear VLBI fringes obtained at the JIVE on baselines between the GRAO and stations at Medicina (Italy), Yebes (Spain), Zelenchukskaya (Russia), and Hartebeesthoek (South Africa) [15]. The Jodrell Bank Centre for Astrophysics, supported by the UK’s STFC/Newton Fund, has developed a new pulsar timing system (Hebe) for the GRAO, with the first pulsar detection made in the C band on 30 April 2017 [10]. The targeted radio continuum flux measurements and emission lines spectroscopy make up most of the single-dish science cases [6]. The GRAO science cases above should be contrasted with those at similar retrofitted facilities elsewhere in the world. Below, we list a few of other such facilities while highlighting the congruence and potential paths for the GRAO and future AVN stations.

In 2001, the National Astronomical Observatory of Japan (NAOJ) was given a 32 m antenna from the KDDI corporation. The NAOJ successfully converted the latter into the Yamaguchi 32 m class radio telescope, in collaboration with the Yamaguchi University and other institutes. The facility was dedicated to (1) use as an element of the Japanese global VLBI network, (2) methanol (6.7 GHz) and water (22 GHz) maser monitoring campaigns, and (3) the education and popularisation of radio astronomy among surrounding communities [16]. Most of these goals are congruent with what is at stake at the GRAO and future AVN stations.

In 2008, an idling telecommunications station with a 32 m parabolic antenna was donated to the Geophysical Institute of Peru. With major support from the National Astronomical Observatory of Japan, the facility was converted into the Sicaya radio telescope. Initially, in the single-dish mode, it was aimed at methanol maser and young stellar objects observations, using an uncooled 6.7 GHz (C band). Joining the VLBI observations remains in sight [17].

Around 2011, a consortium of universities was set up with the goal of using the decommissioned telecommunications infrastructure at the Goonhilly Earth Stations in Cornwall (UK) for astronomical purposes at the L, C, and K bands. The simulations showed that the inclusion of the 30 m class antennas at the Goonhilly Earth Station into e-MERLIN would double the resolving power of the array by increasing the maximum baseline from 217 to 441 km. Resolving the molecular gas at high-redshift with a resolution exceeding that of all but the highest ALMA bands was recognised as a major strength of the intended facility [18]. This illustrates the potential at stake when proper human and financial capital are assembled. One notes with strong interest the increasingly tight working relationship between key members of the consortium above and AVN partners through such initiatives as the DARA.

The 3-year-long conversion, in New Zealand, of the Warkworth 30 m antenna into a radio telescope is probably the most recent example that bears the most relevance to the phased process at the GRAO while also providing foresight on what can be routed beyond retrofitting for future AVN stations. The Warkworth 30 m radio telescope had its “official” First Light on 4 July 2014. By that time, it was already a fully steerable radio telescope operating at the C band for commissioning observations using an uncooled receiver, much like the GRAO today. Already a valuable instrument in its own right for single-dish observations, on 11 December 2014, the first geodetic and astrophysical scientific VLBI experiment was conducted. Although the VLBI fringes checks have been performed at the GRAO, both the full-scale geodetic and astrophysical scientific VLBI remain to be tested at the GRAO. The observatory has access to a 10 Gbit/s connection to the Research and Education Advanced Network New Zealand, providing high-speed data transfers for the e-VLBI. The plans underway include equipping the facility with a cryogenically cooled C-band receiver, an X-band receiver, and a 2 Gbps recording system. For the science case, both the VLBI and single-dish spectral observations in the C band can potentially include spectral line observations of OH maser lines at 6.03 and 6.035 GHz and methanol masers at 6.7 GHz. The addition of S- and X-band receivers would enable the use of the facility for geodetic research [19,20]. The human capital impact has already proven valuable with the radio telescope providing a facility for unparalleled laboratory work and students’ research projects, some of it geared towards design work for the SKA, from New Zealand. Unambiguously, the Warkworth 30 m radio telescope provides a compelling example for what a typical AVN station can be and do, once established.

## 4. Radio Interferometer, *uv*-Coverage, and VLBI

Following the van Cittert–Zernike theorem [21], the output from a two-elements interferometer is a complex measurement; the so-called “visibility’, under specific conditions (e.g., assuming no sampling and other corruption effects), is given by:

(1) $$V\left(u,v,w\right)=\int \int \frac{I(l,m)}{n}e^{-2i\pi (ul+vm+w(n-1))}\mathrm{dldm},$$

where $n^{2}=1-l^{2}-m^{2}$ and I is the apparent sky with coordinates (l,m). The signals received by each of the two-elements interferometer are cross-correlated, either in real time or offline, these cross-correlation products are accumulated during a defined period (the integration time) and at each channel. If the number of antennas is *n*, then the instantaneous number of correlation during the integration time is n(n−1)/2. Because the relative orientation of the antennas and the sources change as the earth rotates, one can take advantage of the earth’s rotation to measure more samples. For an in-depth discussion, we refer the reader to [21,22].

The differential of the spatial frequencies *u*, *v*, and *w* measured in wavelength as a function of the baseline vector with components $L_{x}$, $L_{y}$, $L_{z}$ along the axes of the International Terrestrial Reference Frame (ITRF, a terrestrial coordinate system that provides the coordinates of points on the earth’s surface [23]) is given by:

(2) $$\frac{\partial u}{\partial t}=\frac{\omega_{e}}{\lambda }\left(L_{x}\cos h-L_{y}\sin h\right)$$

(3) $$\frac{\partial v}{\partial t}=\frac{\omega_{e}}{\lambda }\left(L_{x}\sin\delta \sin h+L_{y}\sin\delta \cos h\right)$$

(4) $$\frac{\partial w}{\partial t}=\frac{-\omega_{e}}{\lambda }\left(L_{z}\cos\delta \sin h+L_{y}\cos\delta \cos h\right),$$

where the baseline is tracking a source at declination δ and hour angle *h*, $\omega_{e}=7.2925\times 10^{-5}$ rad·s${}^{-1}$ is the angular velocity of the earth. The uv-coverage is the set of all the projected baseline vectors, (u,v,w) in the Fourier plane or uv-plane. An efficient way to fill the uv-coverage is to add many antenna telescopes together while making use of the earth’s rotation, the frequency coverage, and antennas layout of the interferometer. The more complete the uv-coverage, the better the response of the instrument, and therefore the image quality. In order to achieve a milliarcsecond resolution, the network of antennas requires baselines longer than $10^{4}$ km. Achieving such a high-resolution observation requires a VLBI technique.

### Why the Missing Samples in the EVN + MeerKAT + Kuntunse uv-Coverage?

**Figure 2:**
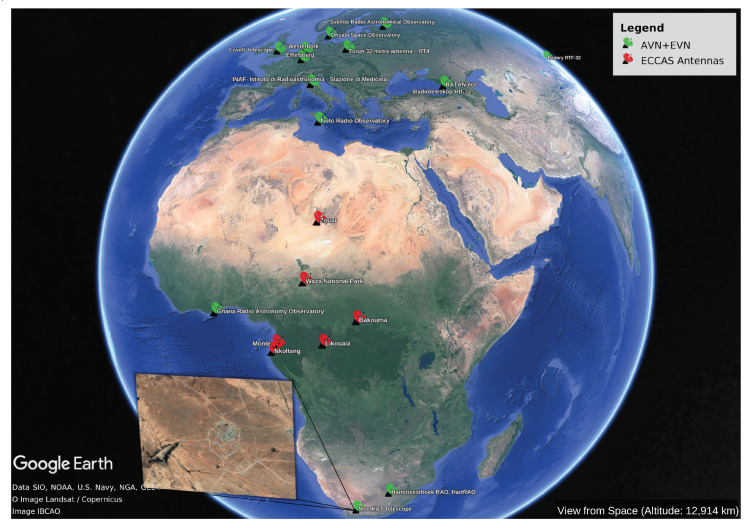
Green points: locations of the Kuntunse antenna in Ghana, the MeerKAT stations in South Africa, and the EVN. Red points: locations of abandoned old telecommunication satellite facilities in the ECCAS region and/or possible sites to build new radio telescopes.

In this section, we discuss the performances of the uv-coverage density of the combined Kuntunse antenna in Ghana with the MeerKAT telescope in South Africa, correlated to the full EVN. The full EVN consists of 12 stations across the globe, i.e., Badary, Effelsberg, Hartebeesthoek, Jodrell Bank, Medicina, Noto, Onsala, Shanghai, Svetloe, Torun, Westerbork, and Zelenchukskaya. Figure 2 shows an African map where the green points are the Kuntunse antenna in Ghana, the MeerKAT telescope in South Africa, and the EVN. There are some stations of the EVN that do not appear on the map (e.g., Shanghai); these are stations that are on the other side of the globe. The 64 antennas of the MeerKAT telescope do not appear all on the map; this is because the antennas are very close to each other, making it difficult to visualise them on a bigger scale. The points in red are some of the locations of the abandoned old telecommunications satellites in the ECCAS countries or locations suitable to build new radio antennas.

**Figure 3:**
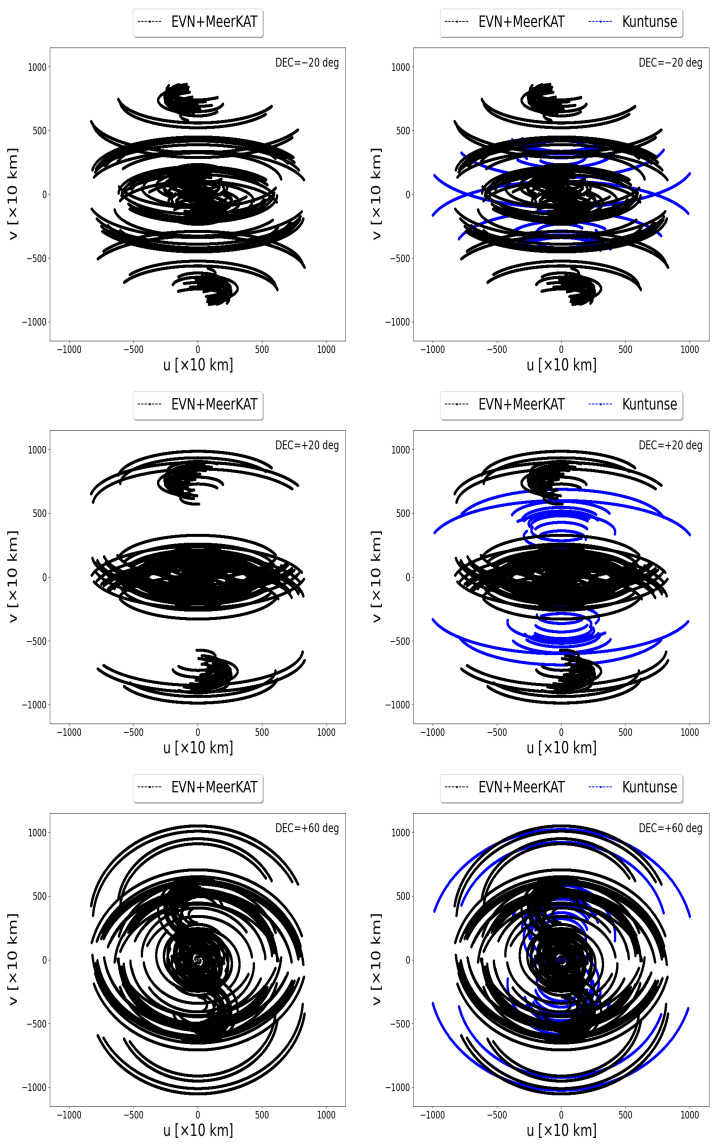
The EVN combined with the MeerKAT telescope (left panels) and the EVN combined with the MeerKAT telescope and the Kuntunse antenna in Ghana (right panels).

Figure 3 shows the uv-coverage of the MeerKAT telescope correlated to the EVN (left panels) and the Kuntunse combined with the MeerKAT telescope and both correlated to the EVN (right panels). These uv-coverages are obtained by a simulation at a frequency of 16 GHz, during a total period of 10 h with 1 s integration time and 16 MHz bandwidth divided into 64 channels. Using the casacore (https://pypi.org/project/python-casacore/, accessed on 15 June 2022), we generate the casa table from the antenna positions specified in the ITRF coordinate system [23] and then we create an empty measurement set using simms (https://github.com/ratt-ru/simms, accessed on 15 June 2022). From the empty measurement set, the uv-coverage is extracted. The uv-coverages are tracking a source at the declination of −20, +20, and +60 deg. It is clearly seen from the correlation between the MeerKAT telescope and the EVN (left panels in Figure 3) that there are missing samples in the middle area. The latter correlation is then combined with the Kuntunse antenna in Ghana which still does not optimally sample these missing areas (see the right panels in Figure 3). In these uv-coverages, the samples from the core are from shorter baselines; these shorter baselines are the internal baselines of the MeerKAT telescope and the EVN. The samples at the outer core are from the longer baselines; these baselines relate the AVN antennas in the northern hemisphere to the MeerKAT telescope and Hartebeesthoek in the southern hemisphere. There are few medium-length and long baselines coming from the correlation between the antennas in the northern hemisphere or southern hemisphere to the Kuntunse antenna in Ghana. The areas sampled by these medium-length and long baselines linked to the Kuntunse antennas are seen in blue from Figure 3, right panels. To fill these missing samples, we need more medium-length baselines. These medium-length baselines can only be obtained if some of the antennas are placed around the equatorial line in Africa. Most of the countries in Africa around the equator are the French-speaking countries or the ECCAS countries. Using simulations, we show in this work that if one were to build radio telescopes and/or convert old abandoned telecommunication satellite antennas to radio telescopes in the ECCAS countries, no matter where these antennas are to be located in each of these countries, this should significantly improve the uv-coverage of the current EVN + MeerKAT + Kuntunse.

## 5. Simulations and Discussion

Two simulations are performed using antennas, as shown in Figure 2. Firstly, to evaluate the performance of the single interferometer array represented by the six antennas of the ECCAS countries (i.e., the antennas with their position marked in red in Figure 2), we generate a measurement set using the procedure described in Section 4.1 and then fill the measurement set with simulated visibilities using MeqTrees [24]. Secondly, using the same simulation tools, we correlate the six ECCAS antennas with the AVN and the EVN and then we simulate the visibilities. To demonstrate the scientific advantages of adding these six antennas to the current VLBI network, for each of the two simulations, we made an image and measure its S/N.

### 5.1. Performance Assessment of the uv-Coverage of the ECCAS Antennas

**Figure 4:**
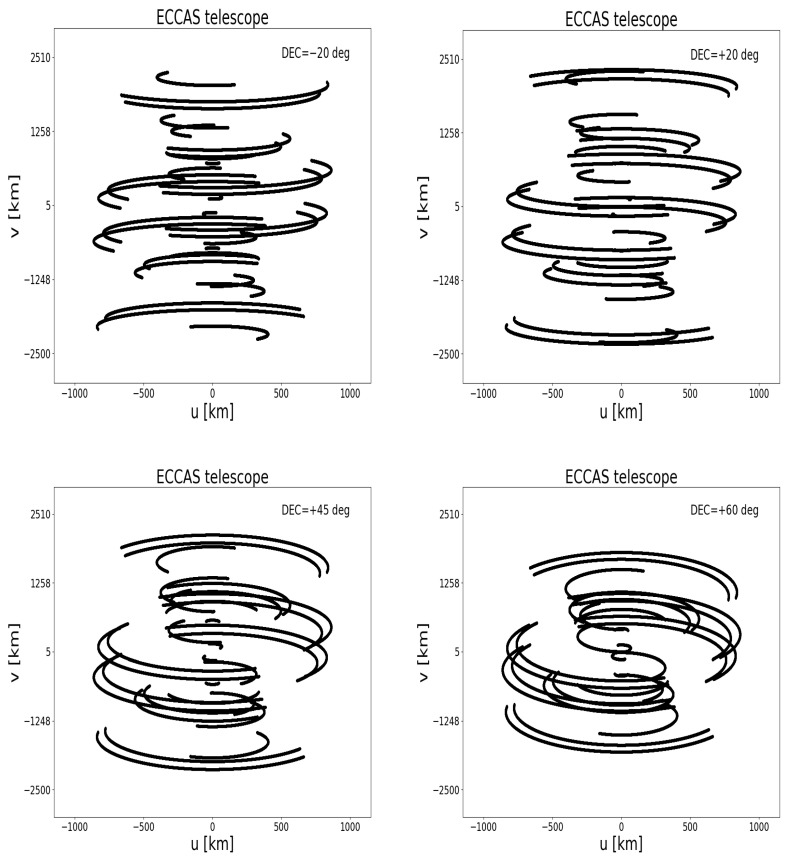
ECCAS antennas uv-coverage at 1.4 GHz at four declinations (−20, +20, +45, and +60 deg), 10 h observation, and 16 MHz total bandwidth showing a lot of holes or gaps.

Figure 4 shows the uv-coverage of the six antennas in the ECCAS countries at 1.4 GHz and for four declinations. This uv-coverage is simulated during 10 h total time with 1 s integration time and a total bandwidth of 16 MHz divided into 64 channels. The positions of the antennas are shown in Figure 2, red points. As expected, the uv-coverage is very poor as the six antennas are spread over a large distance. Each of these antennas can function as a single-dish radio telescope and can perform high-level science, e.g., observing pulsars, masers, and hot gas from the Milky Way or distant galaxies.

### 5.2. Filled uv-Coverage for ECCAS + MeerKAT + EVN + Kuntunse

**Figure 5:**
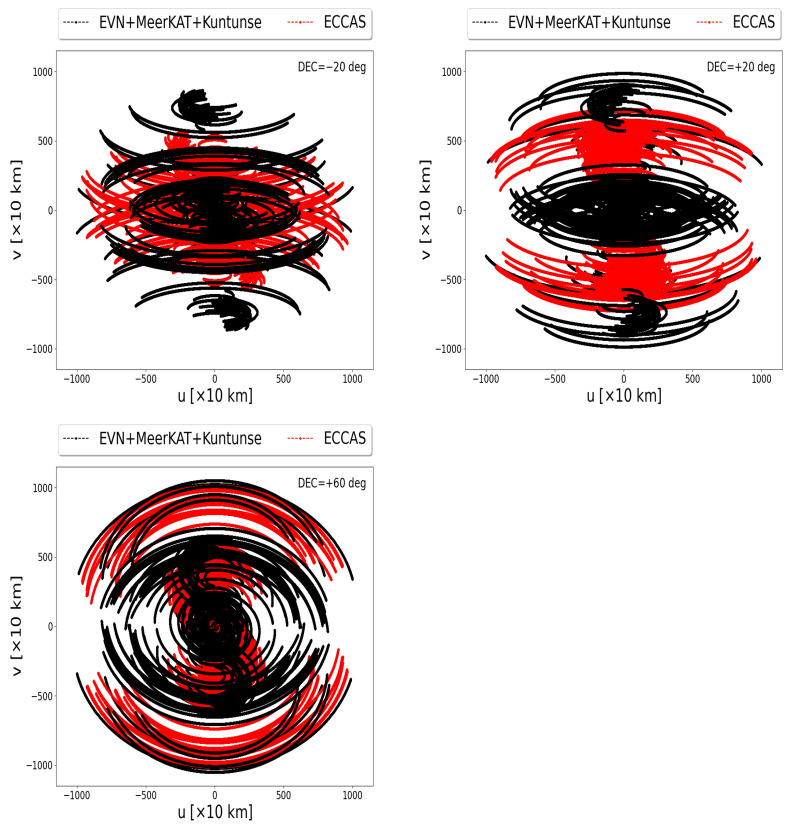
Performance of the global VLBI uv-coverage. The ECCAS antennas are correlated with the Kuntunse antenna, the MeerKAT telescope, and the EVN. The uv-coverage is well-filled because of the extra medium-length baselines that relate the EVN and MeerKAt telescope to the ECCAS antennas.

This time, the MeerKAT telescope, EVN, and Kuntunse are correlated to the ECCAS antennas. The full antennas used in the simulation are shown in Figure 2. The black points in Figure 5 are the data from the EVN, MeerKAT telescope, and Kuntunse antenna while the red points are the data coming from the ECCAS antennas and their correlation to the EVN, MeerKAT telescope, and Kuntunse. We note that while the six ECCAS antennas on their own give poor uv-coverage, as shown in Figure 4, they significantly improve the current uv-coverage of the full VLBI network; the uv-coverage is now well filled because of the extra medium-length baselines that relate the EVN and MeerKAT telescope to the ECCAS antennas. These uv-coverages can even be optimised further if more antennas are added at the equatorial line. Below, using simulated images, we demonstrate that adding these antennas at the equatorial line will improve the S/N and therefore the image quality of the combined radio interferometers.

### 5.3. An Estimate of the Image S/N

In this section, we describe the procedure used to estimate the S/N in our images. As discussed in [25], an approximate of the S/N is given as:

(5) $$S/N∼\frac{A_{\mathrm{flux}}}{C+\sigma },$$

where $A_{\mathrm{flux}}$ is the brightness distribution of a point source, C is the confusion noise, i.e., the unwanted signals that contaminate the field of the source of interest. The root mean square noise σ describes the sensitivity of the instrument and is approximated as:

(6) $$\sigma ∼\frac{\sigma_{\mathrm{vis}}}{\sqrt{n_{\mathrm{ch}}n_{\mathrm{int}}n_{\mathrm{pol}}n_{\mathrm{bal}}}},$$

where $\sigma_{\mathrm{vis}}$ and $n_{\mathrm{ch}}$ are the noise per visibility and the number of channels in the bandwidth, respectively, $n_{\mathrm{int}}$ and $n_{\mathrm{pol}}$ are the number of correlations in time and polarisations, respectively, and $n_{\mathrm{bal}}$ is the total number of baselines. The product $n_{\mathrm{ch}}n_{\mathrm{int}}n_{\mathrm{pol}}n_{\mathrm{bal}}$ is the total number of visibilities in the uv-plane. For an in-depth discussion on how to derive Equation (6), we refer the reader to [25]. During an observation, the confusion noise decreases with the increasing sampled Fourier coefficients in the uv-coverage. Moreover, adding antennas increases the number of baselines which then decreases the root mean square noise. In other words, by adding the ECCAS antennas to the current AVN, we optimise for the unsampled Fourier coefficients in the AVN uv-coverage which then limits the far-field contamination while reducing the dynamic range required to image the source and relaxes the requirements for deconvolving the source. Thus, assuming that $A_{\mathrm{flux}}$ remains constant for both the EVN + MeerKAT + Kuntunse and EVN + MeerKAT + Kuntunse + ECCAS, we see that the S/N will increase for the EVN + MeerKAT + Kuntunse + ECCAS because, by definition, as shown in Equation (6), C and σ are smaller when observing with the EVN + MeerKAT + Kuntunse + ECCAS compared to the EVN + MeerKAT + Kuntunse. To demonstrate the latter, the measurement sets described above are used to simulate the EVN + MeerKAT + Kuntunse and the EVN + MeerKAT + Kuntunse + ECCAS telescopes.

**Figure 6:**
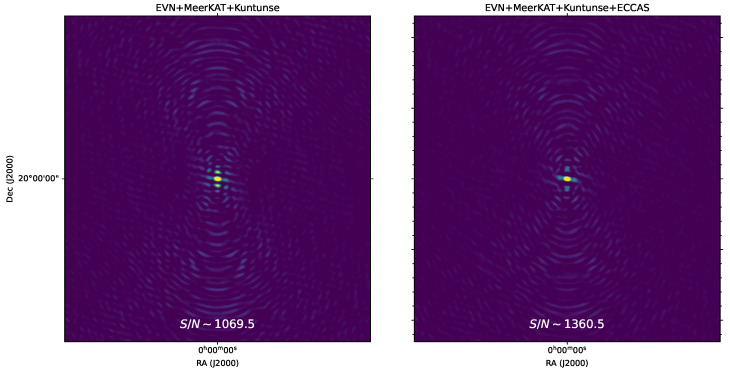
Simulated sky model with two point sources: a 1 Jy point source at the phase centre and a far-field point source with 5 Jy brightness located at 6 arcmin from the phase centre as seen by the EVN + MeerKAT + Kuntunse telescope (left) and by the EVN + MeerKAT + Kuntunse + ECCAS telescope (right) for a simulated observation at 16 GHz. To corrupt the simulation, 1 Jy Gaussian noise per visibility is used. The data are sampled during a total period of 10 h with 1 s integration time and using a total bandwidth of 16 MHz divided into 64 channels. Then, a few pixels are imaged to visualise only the source at the phase centre.

To populate the measurement sets with visibilities, the MeqTrees [24] software is used to simulate a sky model with two point sources: a 1 Jy point source at the phase centre and a far-field point source with 5 Jy brightness located at 6 arcmin from the phase centre. The 1 Jy point source at the phase centre represents our source of interest for which we want to measure its S/N. This source is simulated at the phase centre to limit the amplitude attenuation or the effect of smearing so that one can estimate the $A_{\mathrm{flux}}$ effectively from the simulation. In the VLBI regime, a source at 6 arcmin is already a far-field source and, therefore, can be used to measure the contamination, C, at the phase centre. MeqTrees is used to corrupt the simulated visibility data with 1 Jy Gaussian noise per visibility. The WSclean imager [26] is then used to translate the visibilities to the image using uniform weighting, as shown in Figure 6, where a few pixels are imaged to visualise only the single source at the phase centre. The left panel and the right panel of Figure 6 show the 1 Jy point source as seen by the EVN + MeerKAT + Kuntunse and EVN + MeerKAT + Kuntunse + ECCAS telescopes, respectively. It is easy to see from this result that the point source sidelobes as seen by the EVN + MeerKAT + Kuntunse + ECCAS telescope are lower compared to the sidelobes of the same source as seen by the EVN + MeerKAT + Kuntunse telescope: this is a straightforward result from sampling more Fourier coefficients in the uv-plane. We also note that the resolution of both images in Figure 6 does not vary (i.e., the size or shape of the source main lobe does not change): this is due to adding more antenna telescopes that do not increase the longest Fourier modes and, therefore, preserve the same resolution of the instrument. To estimate the S/N of the source at the phase centre, we adopt the following procedures using uniform weighting:

- The 1 Jy point source at the phase centre is simulated separately (as a single source) for each of the radio interferometers and its amplitude $A_{\mathrm{flux}}$ is measured for each image after cleaning.
- The 5 Jy point source at 6 arcmin from the phase centre is also simulated separately as a single point source. Note that one would expect no signal at the phase centre except the contamination C from this far-field source.
- An empty measurement set (without a source) is simulated for each of the instruments, and then a 1 Jy Gaussian noise per visibility is used to corrupt the measurement sets. The root mean square of the image around the phase centre provides an approximate of σ.

At the end of these simulations, we then estimate the S/N from the measured parameters in Table 1. The EVN + MeerKAT + Kuntunese station gave an S/N of ∼1069.5. By adding the ECCAS station, i.e., the EVN + MeerKAT + Kuntunese + ECCAS combination, the S/N improved significantly, and we measure an S/N of ∼1360.5. This clearly demonstrates the strength of the ECCAS station when combined with the AVN network.

## 6. Conclusions

We conclude that the uv-coverage for a full VLBI observation will improve if a few antennas were to be added in the ECCAS region. The resulting Fourier transform of the uv-coverage compactness will only lead to a low confusion noise limit which is suitable for the high signal-to-noise or dynamic range images requirement. Building or converting old, abandoned satellite telecommunication facilities in the ECCAS region is a guarantee that the science results from these antennas will expand the universities’ international visibility. As part of the VLBI, the scientific community of the ECCAS region will be fully prepared for strong scientific involvement with the SKA.

## Figures and Tables

**Table 1:** Measured parameters used to calculate S/N.

Telescope	$A_{\mathrm{flux}}$	C	σ	S/N
EVN + MeerKAT + Kuntunse	1	0.000767	0.000168	1069.5
EVN + MeerKAT + Kuntunse + ECCAS	1	0.000589	0.000146	1360.5

## Data Availability

Not applicable.
